# Integrated intelligent computing application for effectiveness of Au nanoparticles coated over MWCNTs with velocity slip in curved channel peristaltic flow

**DOI:** 10.1038/s41598-021-98490-y

**Published:** 2021-11-19

**Authors:** Muhammad Asif Zahoor Raja, Mohammad Sabati, Nabeela Parveen, Muhammad Awais, Saeed Ehsan Awan, Naveed Ishtiaq Chaudhary, Muhammad Shoaib, Hani Alquhayz

**Affiliations:** 1 Future Technology Research Center, National Yunlin University of Science and Technology, 123 University Road, Section 3, Douliou, Yunlin, 64002 Taiwan, ROC; 2 Electrical Engineering Department, School of Engineering, Australian College of Kuwait, 13015 Safat Kuwait City, Kuwait; 3 Department of Mathematics, COMSATS University Islamabad, Attock Campus, Attock, Pakistan; 4 Department of Electrical and Computer Engineering, COMSATS University Islamabad, Attock Campus, Attock, 43600 Pakistan; 5 Department of Electrical Engineering, International Islamic University, Islamabad, Pakistan; 6 Department of Computer Science and Information, College of Science in Zulfi, Majmaah University, Al-Majmaah, 11952 Saudi Arabia

**Keywords:** Physics, Fluid dynamics

## Abstract

Estimation of the effectiveness of Au nanoparticles concentration in peristaltic flow through a curved channel by using a data driven stochastic numerical paradigm based on artificial neural network is presented in this study. In the modelling, nano composite is considered involving multi-walled carbon nanotubes coated with gold nanoparticles with different slip conditions. Modeled differential system of the physical problem is numerically analyzed for different scenarios to predict numerical data for velocity and temperature by Adams Bashforth method and these solutions are used as a reference dataset of the networks. Data is processed by segmentation into three categories i.e., training, validation and testing while Levenberg–Marquart training algorithm is adopted for optimization of networks results in terms of performance on mean square errors, train state plots, error histograms, regression analysis, time series responses, and auto-correlation, which establish the accurate and efficient recognition of trends of the system.

## Introduction

Peristalsis of fluid occurs due to dynamic waves generated by compression and expansion of flexible channel walls which extend along length of the channel by infusing and transposing the fluid within it. The mathematical formulation of peristaltic flow initialized with a mechanism developed by Fung and Yih^1^ slotting in laboratory reference frame and Shapiro et al.^2^ for wave frame of reference. Up till now, Ebaid^3^ investigated the behavior of peristaltic transport of a Newtonian fluid for influences of wall slip conditions and magnetic field in an asymmetric channel. Abbasi et al.^4^ inspected Joule heating effects on peristaltic flow through an asymmetric channel having convective boundaries. Abbas et al.^5^ demonstrated uses of MHD peristaltsis of blood having nanoparticles in drug delivery via a discordant channel which is almost authoritative in bio-medical field.

Moreover, the ability of nanoparticles including CNTs to transfigure medical imaging, therapeutics, diagnostics, as well as to perform functional biological progressions because of small size and physical correspondence to biological molecules leads to potential diagnostic and therapeutic applications of nanotechnology in biosciences. Several theoretical and experimental endeavors have been made in this regime^6–10^. Also, evolution and progress in medical diagnostics insistently focused upon the improvements in the nanomaterials which leads to the use of hybrid nanoparticles, a suspension of nanoparticles of more than one material. Rashidi et al.^11^ provide dynamics of Ag–MgO with based fluid water through a channel for MHD convection heat transport with dynamic heaters and coolers. Awais et al.^12^ analytically studied peristaltic rheology of hybrid nanofluid with the effects of induced magnetic field. Qureshi et al.^13^ numerically investigated nanoparticle’s dispersion in MHD liquid considering effects of ion slip and Hall currents. Nawaz and Nazir^14^ discussed the role of multiple nanostructures in existence of magnetic field. Application of dynamics of micropolar hybrid nanofluid based on CNTs with the effects of Newtonian heating is discussed by Ahmad and Nadeem^15^. In present investigation, a nanocomposite containing multi-walled carbon nanotubes (MWCNTs) coated with gold nanoparticles (AuNPs) is studied. MWCNT’s carboxylation has been taken in practice to intensify aqueous system’s solubility. Further, experimental observations revealed that MWCNTs coated with biocompatible metallic nanoparticles improve their colloidal behavior by decreasing their hydrodynamic size^16,17^. Among the metallic nanoparticles, AuNPs are capable of tracking cancer markers in patient’s blood and have been practiced to several contexts involving unscrambling biological tool, drug design, virology, laser-induced therapy, local tissue ablation, and Microwave and Radio Frequency ablation etc. Some experimental studies include articles^18–20^.

Process of fluid flow and heat transfer can be demonstrated with the help of differential equations, solution of which is used to examine the properties of flow variables. Since, an exact solution is the required result, however it is impossible to determine due to some intrinsic difficulties and nonlinearity possessed by differential systems. To cope with this difficulty, deterministic solution procedures are adopted involving approximations, numerical techniques or combination of both^21,22^. Numerical solvers based on deterministic procedure for nonlinear systems including Chebyshev Polynomial Approximations, Variational Iteration Method, Adomian Decomposition Method, Pad´e Approximation Technique, Cubic B-Spline Scaling Functions, Homotopy Perturbation Method, Homotopy Analysis Method, Finite Element Method, Finite Difference Method etc. Major features of these methods are expensive computation, determinism, and a precursor analytical methodology and these procedures have their own limitations and deliver same final outcomes as classical one.

Different from deterministic solvers, the human brain inspired techniques are designed to exhibit super intelligence by recognizing patterns from existing training dataset/behavior in a path which follows sub-paths for final results using a random process or stochastic method. Stochastic numerical approximations depend on artificial intelligence solvers mainly through neural networks, further befitted with deterministic approach and extract the limitations of the classical approaches by taking out the required information by input data. A neural network, a nonlinear statistical analysis method, is a mathematical illustration of the human neural pattern, imitating its “learning” and “generalization” capabilities that’s why neural networks subject to the domain of artificial intelligence. The ability of neural networks to effectively establish a relationship among the input and output variables made it applicable in nanotechnology in estimating the material properties, size, shape, volume fraction, and thermophysical features of nanoparticles. Such machine learning techniques have also been applied in realistic problems such as factor analysis in conjunction, uncertainty analysis, image compression, stock exchange, and Generation of Psuedologs etc. Initially, Monro and Robbins^23^ demonstrated stochastic approximation. Later, Sacha and Varona^24^ studied importance and effectiveness of artificial intelligence in nanotechnology. Regarding applications of artificial intelligence in nanotechnology, modelling and prediction of the biological effects of nanomaterials have been studied by Winklera et al.^25^. Raja et al.^26^ investigated application of stochastic numerical solver for physical problems regarding nanofluidic containing multi-walled carbon nanotubes. Raja et al.^27^ analyzed the MHD slip flow of fluid containing carbon nanotubes with the dynamics of MHD of convective heat transfer by employing intelligent computing strategy. Mehmood et al.^28^ inspected neuro-computing paradigm’s design for nonlinear model of MHD Jaffery–Hamel nanofluid flow. The ability of ANN model to assimilate with large parameters as well as efficient, reliable and accurate to the new information made it a subject of research in different fields including modeling of bimodal drug delivery^29^, models of HIV infection of CD4+ t-cell^30^, prediction of radial size of powdered element^31^, fluidic system representing soft tissues and microvessels model^32^, thermal analysis of fluidic model in porous media^33^, energy consumption prediction^34^, micropolar fluidic models involving permeable walled channel^35^, solution of Bagley–Torvik systems^36^, models arising in astrophysics^37,38^, magnetohydrodynamics^39–41^, models in electric circuits^42^, bioinformatics^43,44^, models in atomic physics^45,46^, models in plasma physics^47,48^, energy^49,50^, power^51,52^ and financial studies^53,54^.

In order to validate the mathematical model of the proposed problem, Levenberg–Marquardt Method (LMM) based backpropagation of neural networks is employed to estimate the flow characteristics for volume fraction of AuNPs with appropriate second order velocity slip and convective thermal conditions with the help of statistical analysis. And, as per the authors knowledge, the present fluidic system has not been investigated by incorporating the strength of Artificial Intelligence (AI) techniques through Levenberg–Marquardt Method (LMM) based backpropagation of neural networks to solve Au nanoparticles concentration in peristaltic flow through a curved channel. This factor inspires the authors to investigate an AI technique to solve the fluidic system represented in Eqs. (13–15). The significant features of proposed computing infrastructure are highlighted as follows:

- A novel computing infrastructure via feed-forward artificial neural networks aided with backpropagation of Levenberg–Marquardt method (LMM) for training/learning, i.e., ANN-LMM, is adopted to find the solution of mathematical model for Au nanoparticles concentration in peristaltic flow through a curved channel.
- The fitness function based on mean square error is developed for the execution of ANN-LMM for prediction of the solution for the proposed fluidic system through targeted data set by means biased input data points of training and unbiased inputs for testing and validation.
- Merit functions representing different scenarios to predict numerical data for velocity and temperature profiles to measure the effect of sundry physical parameters of MWCNT based peristaltic flow model involving gold nanoparticles with different slip conditions are effectively implemented with ANNs-LMM with reasonable precision.
- Performance via convergence, precision and stability of the designed ANNs-LMM for the proposed problem of Au nanoparticle concentration in peristaltic flow through a curved channel is authenticated through histogram studies and regression measures.

## Model formulation

**Figure 1:**
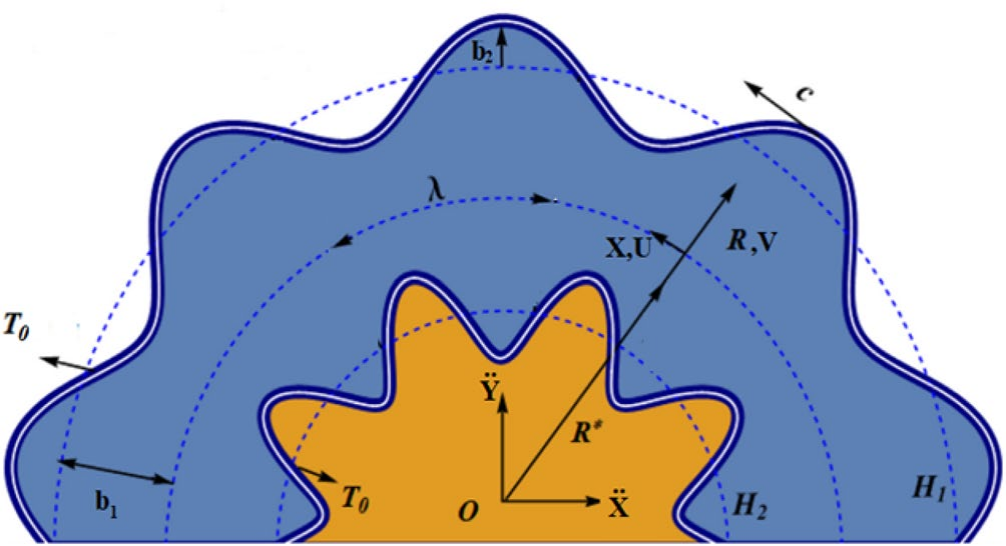
Geometrical interpretation of a symmetrical curved channel.

Consider a curved channel of width *2b*_1_ filled with Newtonian hybrid nanofluid having temperature *T*. The center and radius of curvature of the circle, in which the channel is coiled, be labeled by *O* and ${\text{R}}^{*}$ , accordingly as illustrated in the schematic flow geometry presented in Fig. 1. A curvilinear coordinate system is employed in which *R* is oriented along radial direction while *X* is along the flow direction.

The relation between curvilinear coordinate system and Cartesian coordinate system $\left( {\ddot{{\text{X}}},\ddot{{\text{Y}}}} \right)$ configured at *O* is given by the transformations^55^:

1 $$ \begin{aligned} \ddot{{\text{X}}} & = \left( {{\text{R}}^{ * } + {\text{R}}} \right)\cos \left( {\frac{{\text{X}}}{{{\text{R}}^{ * } }}} \right), \\ \ddot{{\text{Y}}} & = \left( {{\text{R}}^{ * } + {\text{R}}} \right)\sin \left( {\frac{{\text{X}}}{{{\text{R}}^{ * } }}} \right). \\ \end{aligned} $$

Equation of upper boundary in (X, Y) coordinate system is:

2 $$ \ddot{{\text{X}}}^{2} + \ddot{{\text{Y}}}^{2} = \left( {\left( {{\text{R}}^{ * } + {\text{b}}_{1} } \right) + {\text{b}}_{2} \cos \left( {\frac{2\pi }{\lambda }\left( {{\text{R}}^{ * } \tan^{ - 1} \left( {\frac{{\ddot{{\text{Y}}}}}{{\ddot{{\text{X}}}}}} \right) - {\text{ct}}} \right)} \right)} \right)^{2} . $$

Using Eq. (1) into Eq. (2), we have:

3 $$ {\text{R}} = {\text{b}}_{1} + {\text{b}}_{2} \cos \left( {\frac{2\pi }{\lambda }\left( {{\text{X}} - {\text{ct}}} \right)} \right). $$

Thus, for a symmetric channel, equations of upper and lower boundaries are

4 $$ \begin{aligned} {\text{H}}_{1} \left( {{\text{X}},{\text{t}}} \right) & = {\text{b}}_{1} + {\text{b}}_{2} \cos \left( {\left( {\frac{2\pi }{\lambda }} \right)\left( {{\text{X}} - {\text{ct}}} \right)} \right), \\ {\text{H}}_{2} \left( {{\text{X}},{\text{t}}} \right) & = - {\text{b}}_{1} - {\text{b}}_{2} \cos \left( {\left( {\frac{2\pi }{\lambda }} \right)\left( {{\text{X}} - {\text{ct}}} \right)} \right). \\ \end{aligned} $$

where *b*_1_, *b*_2_, *λ* and *c* represents half width and amplitudes of waves in upper and lower halves, wavelength and wave speed, accordingly.

Formulated nonlinear governing model in laboratory frame is given as^55–57^:

5 $$ \frac{{\partial \left\{ {\left( {{\text{R}} + {\text{R}}^{ * } } \right){\text{V}}} \right\}}}{{\partial {\text{R}}}} + {\text{R}}^{ * } \frac{{\partial {\text{U}}}}{{\partial {\text{X}}}} = 0, $$

6 $$ \rho_{{{\text{hnf}}}} \left( {\frac{{\partial {\text{V}}}}{{\partial {\text{t}}}} + V\frac{{\partial {\text{V}}}}{{\partial {\text{R}}}} + \frac{{{\text{R}}^{ * } {\text{U}}}}{{{\text{R}} + {\text{R}}^{ * } }}\frac{{\partial {\text{V}}}}{{\partial {\text{X}}}} - \frac{{{\text{U}}^{2} }}{{{\text{R}} + {\text{R}}^{ * } }}} \right) = - \frac{{\partial {\text{P}}}}{{\partial {\text{R}}}} + \mu_{{{\text{hnf}}}} \left( {\nabla^{2} {\text{V}} - \frac{{2{\text{R}}^{ * } }}{{\left( {{\text{R}} + {\text{R}}^{ * } } \right)^{2} }}\frac{{\partial {\text{U}}}}{{\partial {\text{X}}}} - \frac{{\text{V}}}{{\left( {{\text{R}} + {\text{R}}^{ * } } \right)^{2} }}} \right), $$

7 $$ \rho_{{{\text{hnf}}}} \left( {\frac{{\partial {\text{U}}}}{{\partial {\text{t}}}} + {\text{V}}\frac{{\partial {\text{U}}}}{{\partial {\text{R}}}} + \frac{{{\text{R}}^{ * } {\text{U}}}}{{{\text{R}} + {\text{R}}^{ * } }}\frac{{\partial {\text{U}}}}{{\partial {\text{X}}}} + \frac{{{\text{VU}}}}{{{\text{R}} + {\text{R}}^{ * } }}} \right) = - \frac{{\partial {\text{P}}}}{{\partial {\text{X}}}} + \mu_{{{\text{hnf}}}} \left( {\nabla^{2} {\text{U}} + \frac{{2{\text{R}}^{ * } }}{{\left( {{\text{R}} + {\text{R}}^{ * } } \right)^{2} }}\frac{{\partial {\text{V}}}}{{\partial {\text{X}}}} - \frac{{\text{U}}}{{\left( {{\text{R}} + {\text{R}}^{ * } } \right)^{2} }}} \right), $$

8 $$ \left( {\rho {\text{c}}_{{\text{p}}} } \right)_{{{\text{hnf}}}} \left( {\frac{{\partial {\text{T}}}}{{\partial {\text{t}}}} + {\text{V}}\frac{{\partial {\text{T}}}}{{\partial {\text{R}}}} + \frac{{{\text{R}}^{ * } {\text{U}}}}{{{\text{R}} + {\text{R}}^{ * } }}\frac{{\partial {\text{T}}}}{{\partial {\text{X}}}}} \right) = \kappa_{{{\text{hnf}}}} \left( {\frac{{\partial^{2} {\text{T}}}}{{\partial {\text{R}}^{2} }} + \frac{1}{{{\text{R}} + {\text{R}}^{ * } }}\frac{{\partial {\text{T}}}}{{\partial {\text{R}}}} + \left( {\frac{{{\text{R}}^{ * } }}{{{\text{R}} + {\text{R}}^{ * } }}} \right)^{2} + \frac{{\partial^{2} {\text{T}}}}{{\partial {\text{X}}^{2} }}} \right) + \Phi . $$

The expressions for $\nabla^{2} \,{\text{and}}\,\Phi$ in above equations are:

$$ \begin{aligned} \nabla^{2} & = \frac{{\partial^{2} }}{{\partial {\text{R}}^{2} }} + \frac{1}{{{\text{R}} + {\text{R}}^{ * } }}\frac{\partial }{{\partial {\text{R}}}} + \frac{{{\text{R}}^{ * 2} }}{{\left( {{\text{R}} + {\text{R}}^{ * } } \right)^{2} }}\frac{{\partial^{2} }}{{\partial {\text{X}}^{2} }}, \\ \Phi & = \mu_{{{\text{hnf}}}} \left( {2\left( {\frac{{\partial {\text{V}}}}{{\partial {\text{R}}}}} \right)^{2} + \left( \begin{gathered} \frac{{{\text{R}}^{ * } }}{{{\text{R}} + {\text{R}}^{ * } }}\frac{{\partial {\text{V}}}}{{\partial {\text{X}}}} + \hfill \\ \left( {{\text{R}} + {\text{R}}^{ * } } \right)\frac{\partial }{{\partial {\text{R}}}}\left( {\frac{{\text{U}}}{{{\text{R}} + {\text{R}}^{ * } }}} \right) \hfill \\ \end{gathered} \right)^{2} + 2\left( {\frac{{\text{V}}}{{{\text{R}} + {\text{R}}^{ * } }} + \frac{{{\text{R}}^{ * } }}{{{\text{R}} + {\text{R}}^{ * } }}\frac{{\partial {\text{U}}}}{{\partial {\text{X}}}}} \right)^{2} } \right). \\ \end{aligned} $$

Associated boundary conditions are^58,59^:

9 $$ \begin{aligned} U - U_{w} & = \mu_{hnf} \left( { - \beta_{1} \left( { - \frac{U}{{R + R^{ * } }} + \frac{\partial U}{{\partial R}}} \right) - \beta_{2} \left( {\frac{U}{{\left( {R + R^{ * } } \right)^{2} }} - \frac{1}{{R + R^{ * } }}\frac{\partial U}{{\partial R}} + \frac{{\partial^{2} U}}{{\partial R^{2} }}} \right)} \right), \, \\ \kappa_{hnf} \frac{\partial T}{{\partial R}} & = - h\left( {T - T_{0} } \right),\quad {\text{at}}\,{\text{R}} = H_{1} \left( {X,t} \right), \\ U - U_{w} & = \mu_{hnf} \left( {\beta_{1} \left( { - \frac{U}{{R + R^{ * } }} + \frac{\partial U}{{\partial R}}} \right) + \beta_{2} \left( {\frac{U}{{\left( {R + R^{ * } } \right)^{2} }} - \frac{1}{{R + R^{ * } }}\frac{\partial U}{{\partial R}} + \frac{{\partial^{2} U}}{{\partial R^{2} }}} \right)} \right), \, \\ \kappa_{hnf} \frac{\partial T}{{\partial R}} & = - h\left( {T_{0} - T} \right),\quad {\text{at}}\,{\text{R}} = H_{2} \left( {X,t} \right). \\ \end{aligned} $$

where *β*_1_ and *β*_2_ denote the first and second order slip parameters, *h* is convective heat coefficient while *T*_*0*_ expresses temperature at upper and lower walls and *P* is the pressure. If (R, X, V, U) and (r, x, v, u) denote the coordinates and velocities in the laboratory and wave frame. From laboratory frame to wave frame transformations for steady problem^10,56^ are:

10 $$ \begin{aligned} {\text{r}} & = {\text{R}},{\text{x}} = {\text{X}} - {\text{ct}},{\text{p}}\left( {{\text{r}},{\text{x}}} \right) = {\text{P}}\left( {{\text{R}},{\text{X}} - {\text{ct}}} \right), \\ {\text{v}}\left( {{\text{r}},{\text{x}}} \right) & = {\text{V}}\left( {{\text{R}},{\text{X}} - {\text{ct}}} \right),{\text{u}}\left( {{\text{r}},{\text{x}}} \right) = {\text{U}}\left( {{\text{R}},{\text{X}} - {\text{ct}}} \right) - {\text{c}}. \\ \end{aligned} $$

Introducing stream function *ψ* along with dimensionless variables in laboratory frame^57–59^ as follows:

11 $$ \begin{aligned} {\overline{\text{r}}} & = \frac{{\text{r}}}{{{\text{a}}_{1} }},{\overline{\text{x}}} = \frac{{\text{x}}}{\lambda },\delta = \frac{{{\text{b}}_{1} }}{\lambda },{\text{h}}_{1} = \frac{{{\text{H}}_{1} }}{{{\text{b}}_{1} }},{\text{h}}_{2} = \frac{{{\text{H}}_{2} }}{{{\text{b}}_{1} }},{\overline{\text{v}}} = \frac{{\lambda {\text{v}}}}{{{\text{cb}}_{1} }},{\overline{\text{u}}} = \frac{{\text{u}}}{{\text{c}}},{\overline{\text{p}}} = \frac{{{\text{b}}_{1}^{2} {\text{p}}}}{{{\text{c}}\lambda \mu_{{\text{f}}} }}, \\ {\text{k}} & = \frac{{{\text{R}}^{ * } }}{{{\text{b}}_{1} }},\overline{\beta }_{1} = \frac{{\mu_{{\text{f}}} \beta_{1} }}{{{\text{b}}_{1} }},\overline{\beta }_{2} = \frac{{\mu_{{\text{f}}} \beta_{2} }}{{{\text{b}}_{1}^{2} }},\overline{\psi } = \frac{\psi }{{{\text{b}}_{1} {\text{c}}}},{\overline{\text{v}}} = \frac{{\text{k}}}{{{\overline{\text{r}}} + {\text{k}}}}\frac{{\partial \overline{\psi }}}{{\partial {\overline{\text{x}}}}},{\overline{\text{u}}} = - \frac{{\partial \overline{\underset{\raise0.3em\hbox{$\smash{\scriptscriptstyle\cdot}$}}{\psi } }}}{{\partial {\overline{\text{r}}}}},\theta = \frac{{{\text{T}} - {\text{T}}_{0} }}{{{\text{T}}_{0} }}, \\ \end{aligned} $$

In above equations, $\overline{r},\overline{x},{{\overline{{\text{v}}},}}\overline{u},{\overline{{{\text{p}}}}}\,{\text{and}}\,\theta$ are dimensionless coordinates, velocities, pressure and temperature with *δ* and *k* as wave number and radius of curvature. Non dimensional governing model for the above-mentioned quantities along with small wave number and low Reynolds number approach is:

12 $$ - \frac{{\text{k}}}{{{\overline{\text{r}}} + {\text{k}}}}\frac{{\partial {\overline{\text{p}}}}}{{\partial {\overline{\text{x}}}}} + \frac{1}{{{\text{A}}_{1} }}\left( { - \frac{{\partial^{3} \overline{\psi }}}{{\partial {\overline{\text{r}}}^{3} }} - \frac{1}{{{\overline{\text{r}}} + {\text{k}}}}\frac{{\partial^{2} \overline{\psi }}}{{\partial {\overline{\text{r}}}^{2} }} - \frac{{\left( {1 - \frac{{\partial \overline{\psi }}}{{\partial {\overline{\text{r}}}}}} \right)}}{{\left( {{\overline{\text{r}}} + {\text{k}}} \right)^{2} }}} \right) = 0, $$

13 $$ \frac{{\partial {\overline{\text{p}}}}}{{\partial {\overline{\text{r}}}}} = 0, $$

By eliminating pressure, Eq. (12) simplified in the form:

14 $$ \left( { - \left( {{\overline{\text{r}}} + {\text{k}}} \right)\frac{{\partial^{4} \overline{\psi }}}{{\partial {\overline{\text{r}}}^{4} }} - 2\frac{{\partial^{3} \overline{\psi }}}{{\partial {\overline{\text{r}}}^{3} }} + \frac{1}{{\left( {{\overline{\text{r}}} + {\text{k}}} \right)}}\frac{{\partial^{2} \overline{\psi }}}{{\partial {\overline{\text{r}}}^{2} }} + \frac{{\left( {1 - \frac{{\partial \overline{\psi }}}{{\partial {\overline{\text{r}}}}}} \right)}}{{\left( {{\overline{\text{r}}} + {\text{k}}} \right)^{2} }}} \right) = 0, $$

15 $$ {\text{A}}_{6} \left( {\frac{{\partial^{2} \theta }}{{\partial {\overline{\text{r}}}^{2} }} + \frac{1}{{{\overline{\text{r}}} + {\text{k}}}}\frac{\partial \theta }{{\partial {\overline{\text{r}}}}}} \right) + \frac{{\text{k}}}{{{\overline{\text{r}}} + {\text{k}}}}\frac{{{\text{Br}}}}{{{\text{A}}_{1} }}\left( { - \frac{{\partial^{2} \overline{\psi }}}{{\partial {\overline{\text{r}}}^{2} }} - \frac{1}{{{\overline{\text{r}}} + {\text{k}}}}\left( {1 - \frac{{\partial \overline{\psi }}}{{\partial {\overline{\text{r}}}}}} \right)} \right)^{2} = 0, $$

Corresponding boundary conditions are written as:

16 $$ \begin{aligned} & \overline{\psi } = - \frac{{\text{F}}}{2}, - \frac{{\partial \overline{\psi }}}{{\partial {\overline{\text{r}}}}} + \frac{{\overline{\beta }_{1} }}{{{\text{A}}_{1} }}\left( { - \frac{{\partial^{{2}} \overline{\psi }}}{{\partial {\overline{\text{r}}}^{{2}} }} - \frac{1}{{{\overline{\text{r}}} + {\text{k}}}}\left( {1 - \frac{{\partial \overline{\psi }}}{{\partial {\overline{\text{r}}}}}} \right)} \right) + \frac{{\overline{\beta }_{2} }}{{{\text{A}}_{1} }}\left( { - \frac{{\partial^{{3}} \overline{\psi }}}{{\partial {\overline{\text{r}}}^{{3}} }} + \frac{1}{{{\overline{\text{r}}} + {\text{k}}}}\frac{{\partial^{{2}} \overline{\psi }}}{{\partial {\overline{\text{r}}}^{{2}} }} + \frac{1}{{\left( {{\overline{\text{r}}} + {\text{k}}} \right)^{2} }}\left( {1 - \frac{{\partial \overline{\psi }}}{{\partial {\overline{\text{r}}}}}} \right)} \right) = 0, \\ & \overline{\psi } = \frac{{\text{F}}}{2}, - \frac{{\partial \overline{\psi }}}{{\partial {\overline{\text{r}}}}} - \frac{{\overline{\beta }_{1} }}{{{\text{A}}_{1} }}\left( { - \frac{{\partial^{{2}} \overline{\psi }}}{{\partial {\overline{\text{r}}}^{{2}} }} - \frac{1}{{{\overline{\text{r}}} + {\text{k}}}}\left( {1 - \frac{{\partial \overline{\psi }}}{{\partial {\overline{\text{r}}}}}} \right)} \right) - \frac{{\overline{\beta }_{2} }}{{{\text{A}}_{1} }}\left( { - \frac{{\partial^{{3}} \overline{\psi }}}{{\partial {\overline{\text{r}}}^{{3}} }} + \frac{1}{{{\overline{\text{r}}} + {\text{k}}}}\frac{{\partial^{{2}} \overline{\psi }}}{{\partial {\overline{\text{r}}}^{{2}} }} + \frac{1}{{\left( {{\overline{\text{r}}} + {\text{k}}} \right)^{2} }}\left( {1 - \frac{{\partial \overline{\psi }}}{{\partial {\overline{\text{r}}}}}} \right)} \right) = 0, \\ & \frac{\partial \theta }{{\partial {\overline{\text{r}}}}} + {\text{Bi}}\theta = 0,\,{\text{at}}\,{\overline{\text{r}}} = {\overline{\text{h}}}_{1} = 1 + a^{\prime}\cos \left( {{\overline{\text{x}}}} \right), \\ & \frac{\partial \theta }{{\partial {\overline{\text{r}}}}} - {\text{Bi}}\left( {\theta - 1} \right) = 0,\,{\text{at}}\,{\overline{\text{r}}} = {\overline{\text{h}}}_{2} = - 1 - a^{\prime}\cos \left( {{\overline{\text{x}}}} \right). \\ \end{aligned} $$

Pertinent parameters on the above system including Prandtl number (*Pr*), Brinkman number (*Br*), Biot number (*Bi*), Reynolds number (*Re*), and amplitude ratio parameter (a′) are mathematically expressed as:

17 $$ {\text{ Pr}} = \frac{{\left( {\mu {\text{c}}_{{\text{p}}} } \right)_{{\text{f}}} }}{{\kappa_{{\text{f}}} }},{\text{Br}} = \frac{{\mu_{{\text{f}}} {\text{c}}^{2} }}{{\kappa_{{\text{f}}} \left( {{\text{T}}_{0} } \right)}},{\text{Bi}} = \frac{{{\overline{\text{h}}\text{b}}_{1} }}{{\kappa_{{\text{f}}} }},{\text{Re}} = \frac{{{\text{b}}_{1} {\text{c}}\rho_{{\text{f}}} }}{{\mu_{{\text{f}}} }},{\text{a}}^{\prime} = \frac{{{\text{b}}_{2} }}{{{\text{b}}_{1} }}. $$

## Methodology

**Figure 2:**
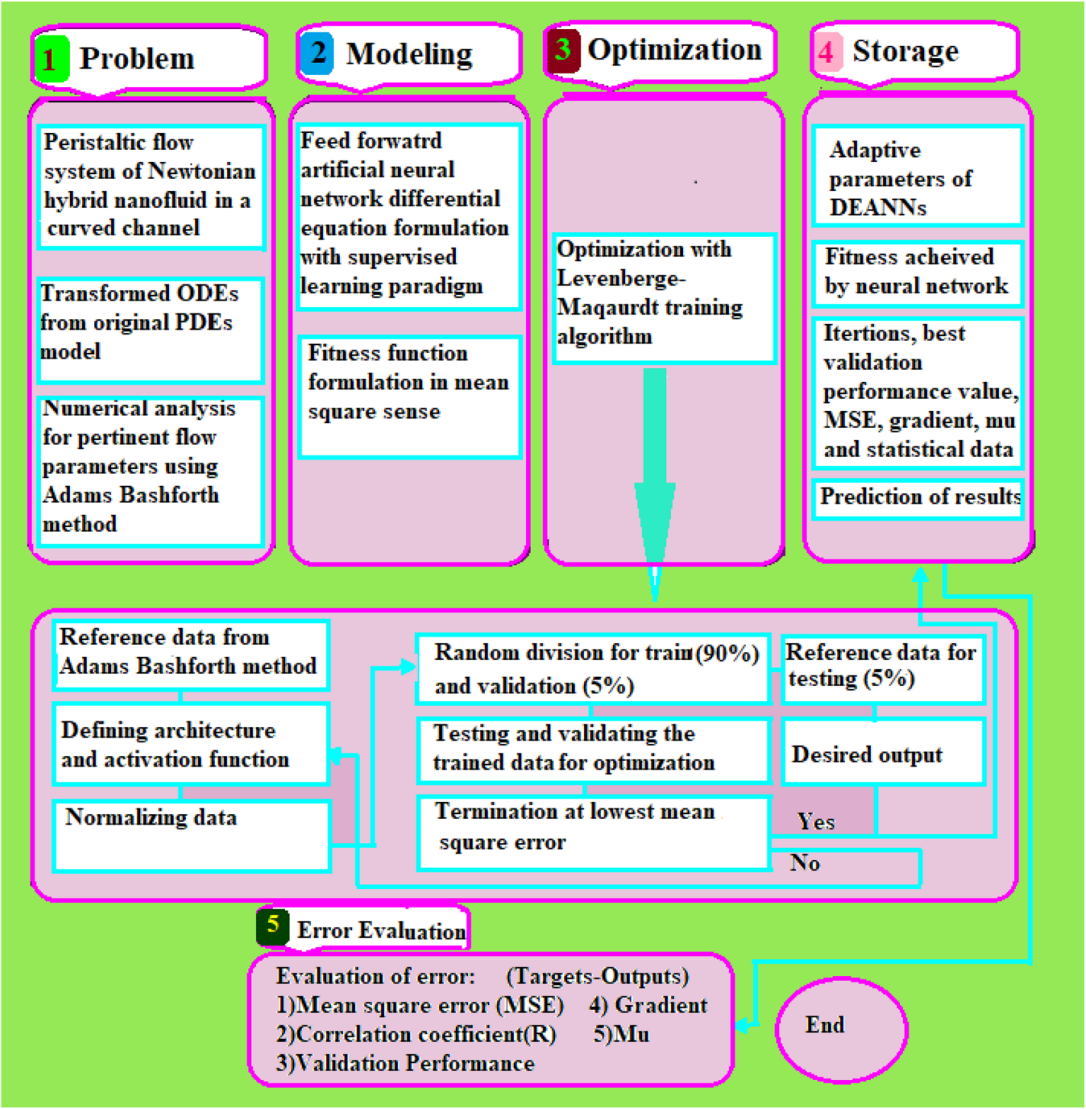
Schematic of proposed methodology.

The methodology adopted in this study is presented here in terms of reference numerical solutions, neural networks modeling and training with Levenberg–Marquardt algorithm as graphically depicted in Fig. 2. The methodology is systematically represented with necessary detail in terms of process block structure consist of problem, modeling optimization and storage steps.

The reference numerical solutions are determined by employing the Adams predictor corrector method^60–62^ to solve the system of Eqs. (13–15) by using boundary conditions as given in Eq. (16). In this numerical approach, predictor solution is predicted first and then corrector is used to calculate the accurate solution by using already predicted solution. Considering the Eqs. (13–15) for velocity $u$ and temperature $\theta$ as:

18 $$ \begin{aligned} \frac{du}{{dr}} & = v(r,u),\quad u(r_{0} ) = u_{0} , \\ \frac{d\theta }{{dr}} & = v(r,\theta ),\quad \theta (r_{0} ) = \theta_{0} . \\ \end{aligned} $$

To derive two-step predictor formula for first equation in (18), integration gives^62^:

19 $$ u(n + 1) = u\left( n \right) + \int_{{r_{n} }}^{{r_{n + 1} }} {v\left( {r,u} \right)dr.} $$

For $v(r,u)$ a linear polynomial, two points $v\left( {n - 1} \right){\text{ and }}v\left( {\text{n}} \right)$ are used and expression is:

20 $$ v\left( {r,u} \right) = v\left( {r_{n} ,u_{n} } \right) + \frac{{v(r_{n} ,u_{n} ) - v(r_{n - 1} ,u_{n - 1} )}}{{r_{n} - r_{n - 1} }}. $$

Then, using above expression in Eq. (20), we have two-step predictor formula as

$$ D_{n + 1} = u_{n} + \frac{h}{2}\left( {3v(r_{n} ,u_{n} ) - v(r_{n - 1} ,u_{n - 1} )} \right). $$

Similarly, for second equation

$$ D_{n + 1} = \theta_{n} + \frac{h}{2}\left( {3v(r_{n} ,\theta_{n} ) - v(r_{n - 1} ,\theta_{n - 1} )} \right), $$

Adams–Bashforth two-step corrector formulas are given by:

$$ \begin{aligned} u_{n + 1} & = u_{n} + \frac{h}{2}(v(r_{n + 1} ,D_{n + 1} ) + v(r_{n} ,u_{n} )), \\ \theta_{n + 1} & = \theta_{n} + \frac{h}{2}(v(r_{n + 1} ,D_{n + 1} ) + v(r_{n} ,\theta_{n} )). \\ \end{aligned} $$

**Figure 3:**
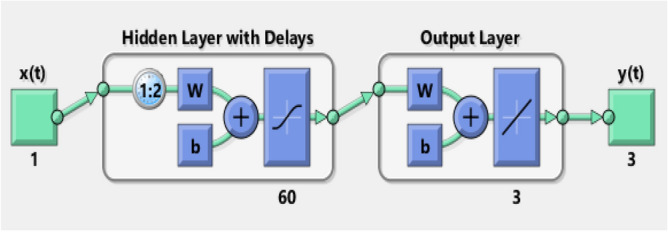
Neural network model for selected architecture.

The dataset generated by Adams predictor corrector method is used in neural networks structure in terms of represented with three layers; input layer, hidden layer and output layer based on a supervised learning approach with a back propagation with Levenberg–Marquart algorithm, while the number of neurons ranging can be taken between 50 and 80 with structure as shown in in Fig. 3.

Training data is acquired from governing model using Adams Bash forth method. Reference data is randomly divided into three groups i.e., one set of example can set training (90%), validation (5%) and testing (5%). Input and the corresponding target provided to the ANN model is received by neurons which combine them, perform a nonlinear operation on the result in the hidden layer, and then outputs appear in output layer. Weighted summation of inputs is added with biases in the hidden layer which is transferred then using hyperbolic tangent sigmoid function as an activation function and can be calculated mathematically as^63^:

$$ \sum\limits_{j = 1}^{r} {w_{jk} } p_{j} + b_{k} ,\,\,n_{k} = \frac{2}{{1 + e^{ - 2z} }} - 1. $$

In which, $w_{jk}$ represents the weights of *jth* neuron in previous layer to the *kth* neurons and *kth* neuron is denoted by $n_{k} .$ Levenberg–Marquardt training algorithm is used to fine tune the weights and biases in the networks to minimize the error, and obtain a high performance of accurate solution. The procedural step of Levenberg–Marquardt training is presented in Fig. 2 optimization block. Finally, the output activation function is a linear function mathematically represented as:

$$ f(x) = x + b $$

Learning/retrain operation is continuous execute as the desire set level of error or fitness is achieved, and neural network performances are then evaluated to predict the model efficacy. One set of the architecture analysis of a neural network model is represented in Fig. 3.

Different error and accuracy definitions are proposed to assess neural network model. The performance operators are given as follows^63,64^

$$ MSE = \frac{{\sum\nolimits_{i = 1}^{N} {\left( {p_{i} - y_{i} } \right)^{2} } }}{N},\quad {\text{and}}\quad R = 1 - \frac{{\sum\nolimits_{i = 1}^{N} {\left( {p_{i} - y_{i} } \right)} }}{{\sum\nolimits_{i = 1}^{N} {\left( {p_{i} - \overline{y}_{i} } \right)} }}. $$

where *p*_*i*_ is the predicted value and $y_{i}$ is the corresponding targeted value, $\overline{y}_{i}$ represents the average of targeted value. Values of *R* closer to 1 and lower MSE values are representative of more reliable and accurate prediction.

The software packages used in implementation of the design methodology consist of two different programs. Firstly, we used ‘NDSolve’ routine with algorithm/method ‘adams’ with automatics settings of stoppage and accuracy goal parameters for finding the numerical solution of systems representing Au nanoparticles coated over MWCNTs with second order velocity slip in curved channel peristaltic flow using Matheamtica Software package for solution of differential systems. While in the second phase, ‘nftool’ routine with appropriate setting of hidden neurons in neural network toolbox of MATLAB software package is used for implementation of the networks optimized/trained/learned with backpropagation of Liverberg–Marquardt for getting the approximate solutions of system model.

## Results and discussion

In this section, results of proposed study ANN-LMM along with the reference solutions as per procedure provided in the last section are presented for fluidic model of peristaltic flow through a curved channel to predict the flow and heat transfer characteristics.

In the presented simulated study, neural networks are exploited to peristaltic flow through a curved channel to predict the flow and heat transfer characteristics. Numerical solutions, as well as, neural network estimated results are figured out and explained. The physical parameter settings of different variation are tabulated in Table 1. Case study 1 represents the variable of velocity profile with three scenarios. Scenarios 1, 2 and 3 considered variation in values of volume fraction of gold nanoparticles *ϕ*_2_, first order slip parameter *β*_1_ and second order slip parameter *β*_2,_ respectively, which, each scenario has four corresponding cases as mentioned in Table 1. Similarly, case study 2, defines for fluid temperature with two scenarios in which variation in magnitudes of *ϕ*_2_ and *Bi* are considered with four different cases.

**Table 1:** Physical parameter settings of the model.

Case study number	Scenario number	Cases index			
		1	2	3	4
1	1:$\upphi _{2}$	0.0	0.2	0.4	0.6
	2:$\upbeta _{1}$	0.0	0.03	0.06	0.09
	3:$\upbeta _{2}$	0.0	0.02	0.04	0.06
2	1:$\upphi _{2}$	0.0	0.03	0.06	0.09
	2:${\text{Bi}}$	0.5	0.6	0.7	0.8

Before providing the results of proposed algorithm ANN-LMM, first the necessary elaborative information/criteria/justification for selection of appropriate inputs samples, hidden neurons as well as training, testing and validations percentages is presented.

The dataset is formulated with the help of Adams numerical solver for all four variations of each scenario of both case studies of the systems model for inputs between − 1.4 and 1.4 with step size 0.015, i.e., 561 data points or sample for each case. The dataset constructed for each case is used for finding the approximate solution of the problem, if we increase the data points the accuracy of the algorithm increase but then you need more hidden neurons in the model to accurate neural networks modeling. So, increase the number of data points will increase the accuracy but at the cast of more computational requirement. After exhaustive experimentation and keeping in view of reasonable compromise between the accuracy of the model and complexity, 561 data points are selected for each case of the system model. The selection of the appropriate neurons for neural network structure is always bit complex procedure. Normally, the number of the neurons in the neural network structure are determined on the basis of the tradeoff between accuracy of model on the training data points. i.e., biased inputs without prior knowledge of original/exact target and level of precision on the testing data inputs, i.e., unbiased inputs with no information of targets. We have extensively performed the simulation study with setting of the neurons between 10, 20 or 30, a relatively a low level of accuracy in training, testing and validation data points is achieved by the proposed methodology with the no noticeable difference in performance for training, testing and validation inputs. However, similarly if increasing the neurons around 300 or more, then results are better for training data inputs while no improvement for testing data inputs. Additionally, with the increase of hidden neurons in neural network structure the computational complexity also increases considerably with some benefit of precision. Therefore, considering both options (accuracy and complexity) after extensive simulation studies, we set 60 number of hidden neurons for our numerical experimentation for solving the model presented in Eqs. (14–16).

We have conducted the experimentations with different combination of the testing, validation and training arbitrary selected data samples, i.e., 70%, 50% and 90% training, 15%, 25% and 5% testing, and 15%, 25% and 5% validation. observations/finding/remarks.

The 70% training, 15% testing and 15% validation, results are consistently obtained and convergent but with low level of the accuracy on the basis of MSE, mean square deviation from the reference numerical solution, in the range of 10^–06^ to 10^–07^ and 10^–05^ to 10^–06^ for testing and validations inputs, while in case of 50% training, 25% testing and 25% validation, results of the training are in 10^–05^ to 10^–06^ but validation and testing accuracy decrease considerable between10^–01^ to 10^–02^, and accordingly, in case of 90% training, 5% testing and 5%. The accuracy of testing inputs is found in the range of 10^–09^ to 10^–10^, while testing and validations also lies mostly in the range of 10^–08^ to 10^–09^. The results provided here are based on 60 hidden neurons, while small change of number of neurons in neural network structure have no bit impact on the performance.

Keeping in view of all these simulations studies, rest of the study is presented selecting 60 hidden neurons, 561 input or target instances/samples, 90% training and 5% testing and validation, for proposed computing scheme.

### Numerical results

**Figure 4:**
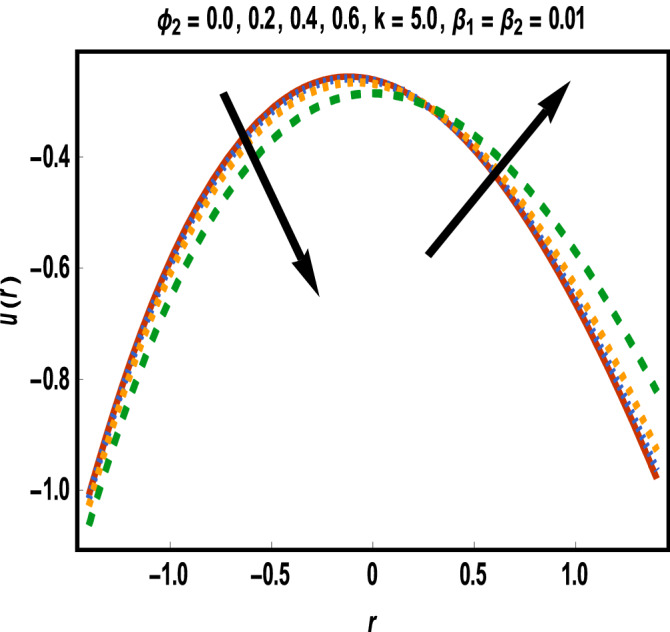
Results of case study 1: scenario 1 (*ϕ*_2_).

**Figure 5:**
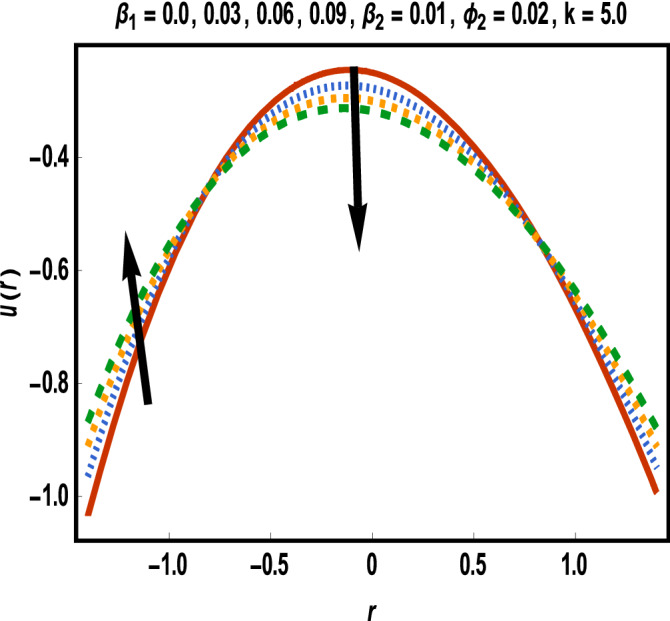
Results of case study 1: scenario 2 (*β*_1_).

**Figure 6:**
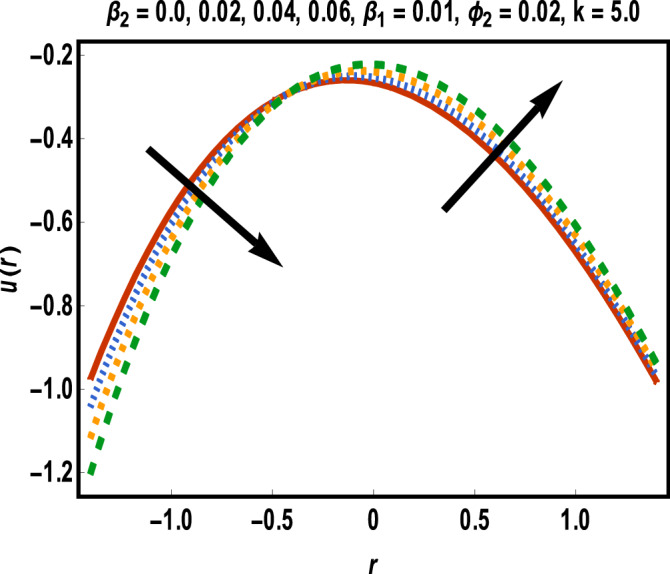
Results of case study 1: scenario 3 (*β*_2_).

**Figure 7:**
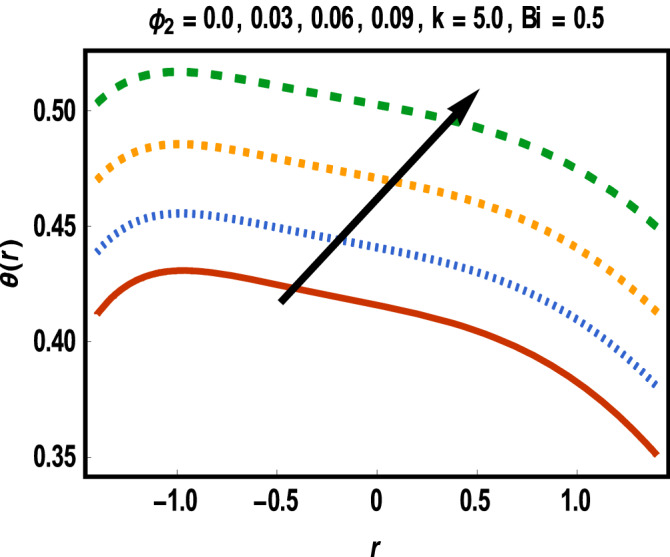
Results of case study 2: scenario 1 (*ϕ*_1_).

**Figure 8:**
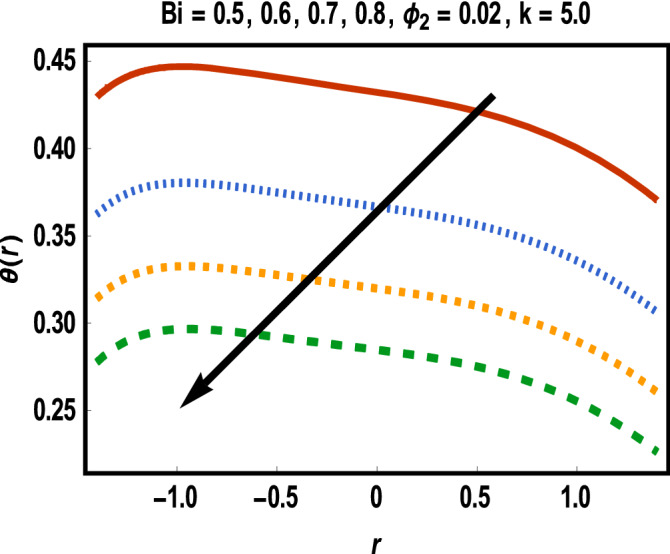
Results of case study 2: scenario 2 (Bi).

Numerical results for reference solutions using Adams Bash forth technique for all cases of each scenario are plotted in Figs. 4, 5, 6, 7 and 8.

In Fig. 4, effect of gold nanoparticle’s volume fraction on fluid velocity is examined. It is evident from graph that maximum value of velocity occurs near the center of the channel. The velocity is increasing near the upper wall whereas an opposite trend is seen closed to the lower wall. This trend may return to an increasing shear stress caused by addition of more gold nanoparticles in lower half. Velocity of fluid shows a reduction in the central part of channel while it enhances near the walls towards variation in *β*_1_ as displayed in Fig. 5.

Increment in values of *β*_1_ represents more slippage at the boundary of surface which decrease the resistive forces and fluid velocity is unaffected by surface motion. Moreover, variation in values of *β*_2_ leads to accelerate the velocity in the vicinity of the upper channel and an opposite trend is observed for lower half. This fact is depicted in Fig. 6. Variational trend of temperature of hybrid nanofluid against $\phi_{2}$ and *Bi* is portrayed in Figs. 7 and 8*.*

It is noticed that an increment in volume fraction of gold nanoparticles for fixed concentration of MWCNTs causes temperature of fluid to upgrade. Clearly, the presence of nanoparticles near cancerous tissues produces thermal energy which increases more due to redistribution of nanoparticles within the channel under peristalsis. Thus, more heated gold nanoparticles near the cancer’s cells directly kill them. Correspondingly, temperature is reduced for increment in values of Bi throughout the channel width. In several cases, Biot number with small values results in uniform distribution of temperature in fluid while some irregularity achieves for Bi larger than 0.1 and thus large values of Biot number are considered***.***

### Evaluation of the networks based stochastic approach

To examine the performance of estimated results obtained by proposed neural network for reference datasets having 561 samples of 3 elements generated through Adams method for each case The analysis based error evaluation including plots of validation performance, training state, error-histograms, time-series response curves, linear regression, and correlation for training, validation and test phases are carried out for different cases of all scenarios with brief interpretations.

#### Case Study 1

**Figure 9:**
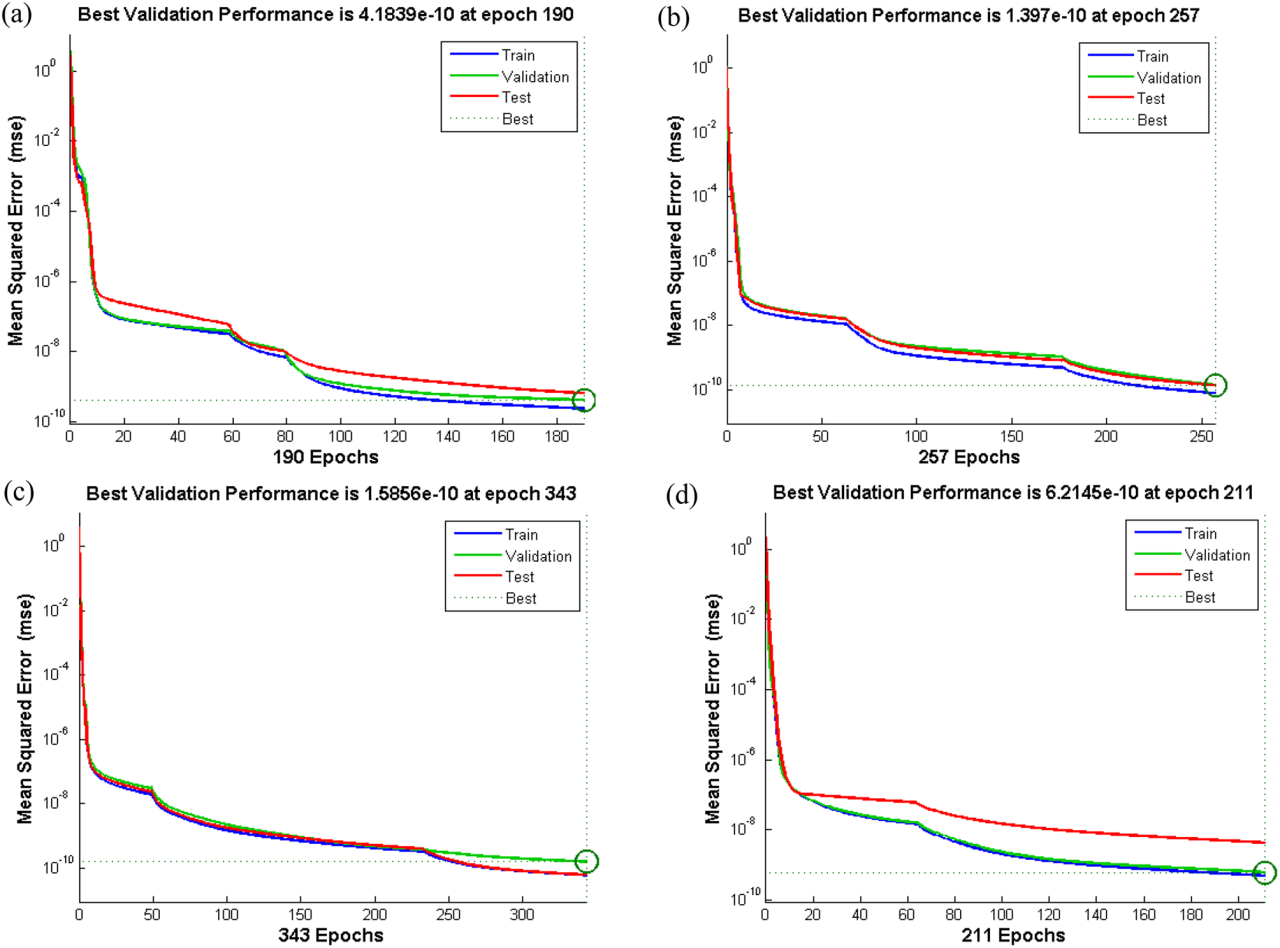
Performance curve of case study 1: scenario1 (**a**) case 1, (**b**) case 2, (**c**) case 3 and (**d**) case 4.

**Figure 10:**
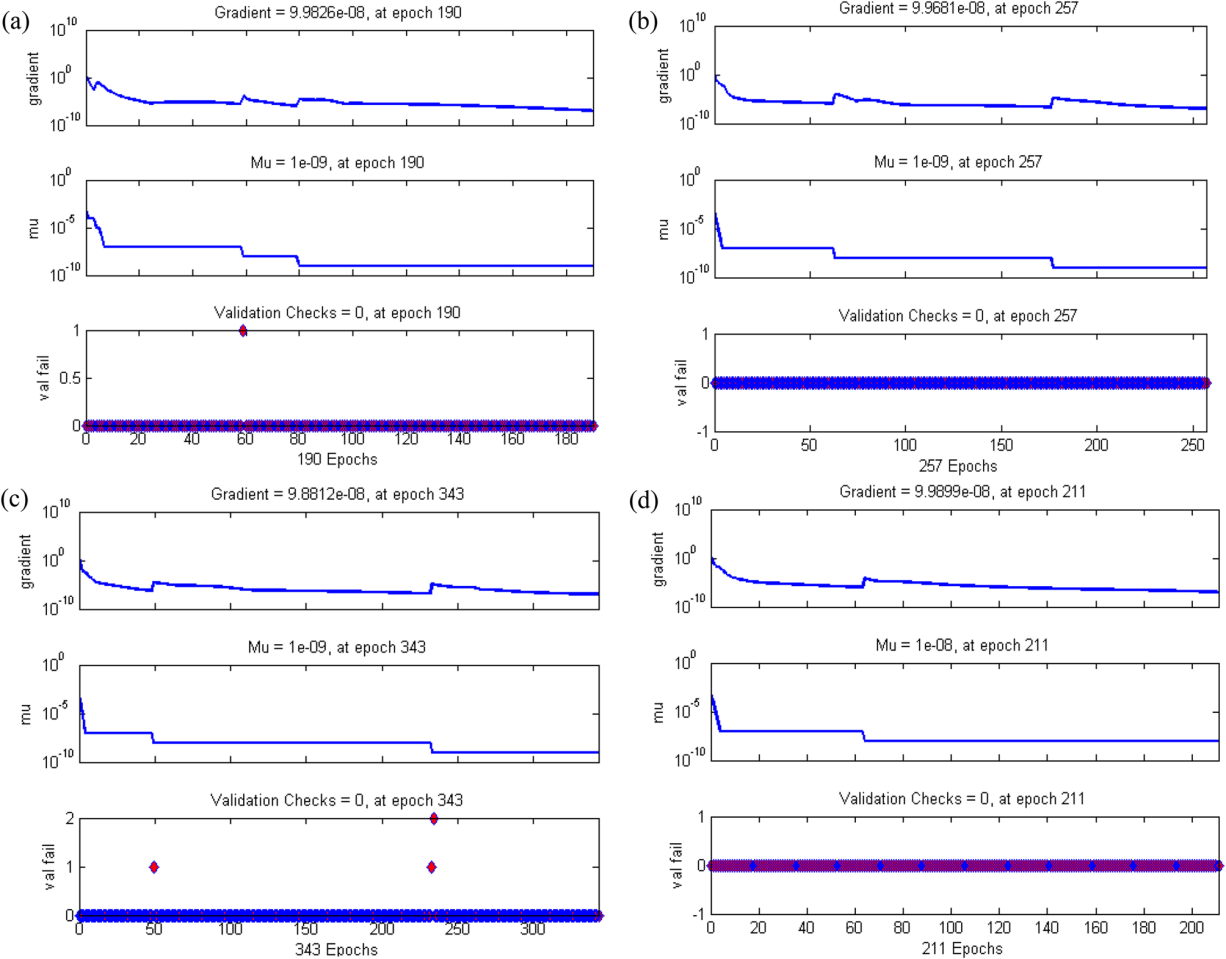
Plot training state of case study 1: scenario 1 (**a**) case 1, (**b**) case 2, (**c**) case 3 and (**d**) case 4.

**Figure 11:**
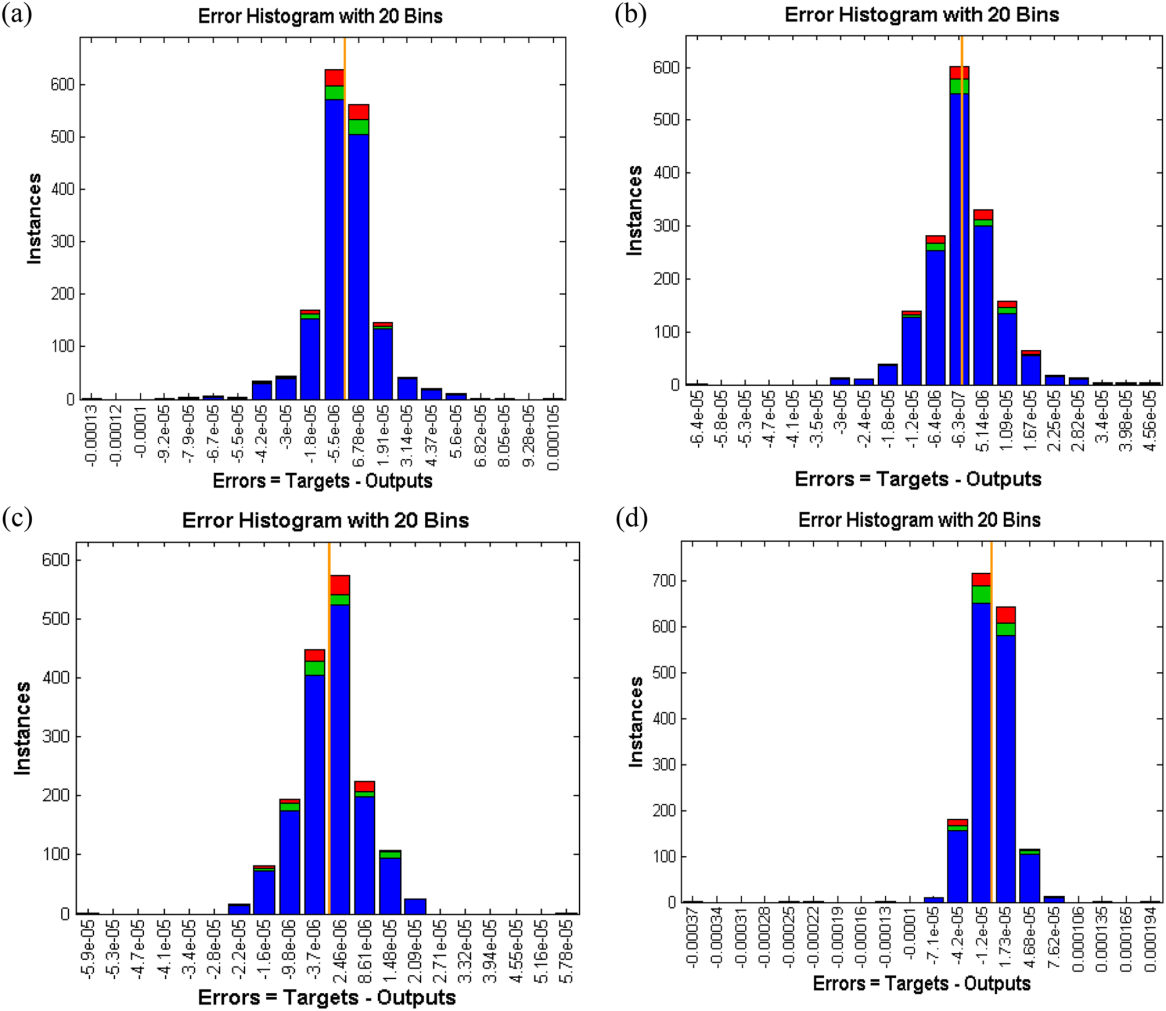
Error histogram of case study 1: scenario 1 (**a**) case 1, (**b**) case 2, (**c**) case 3 and (**d**) case 4.

**Figure 12:**
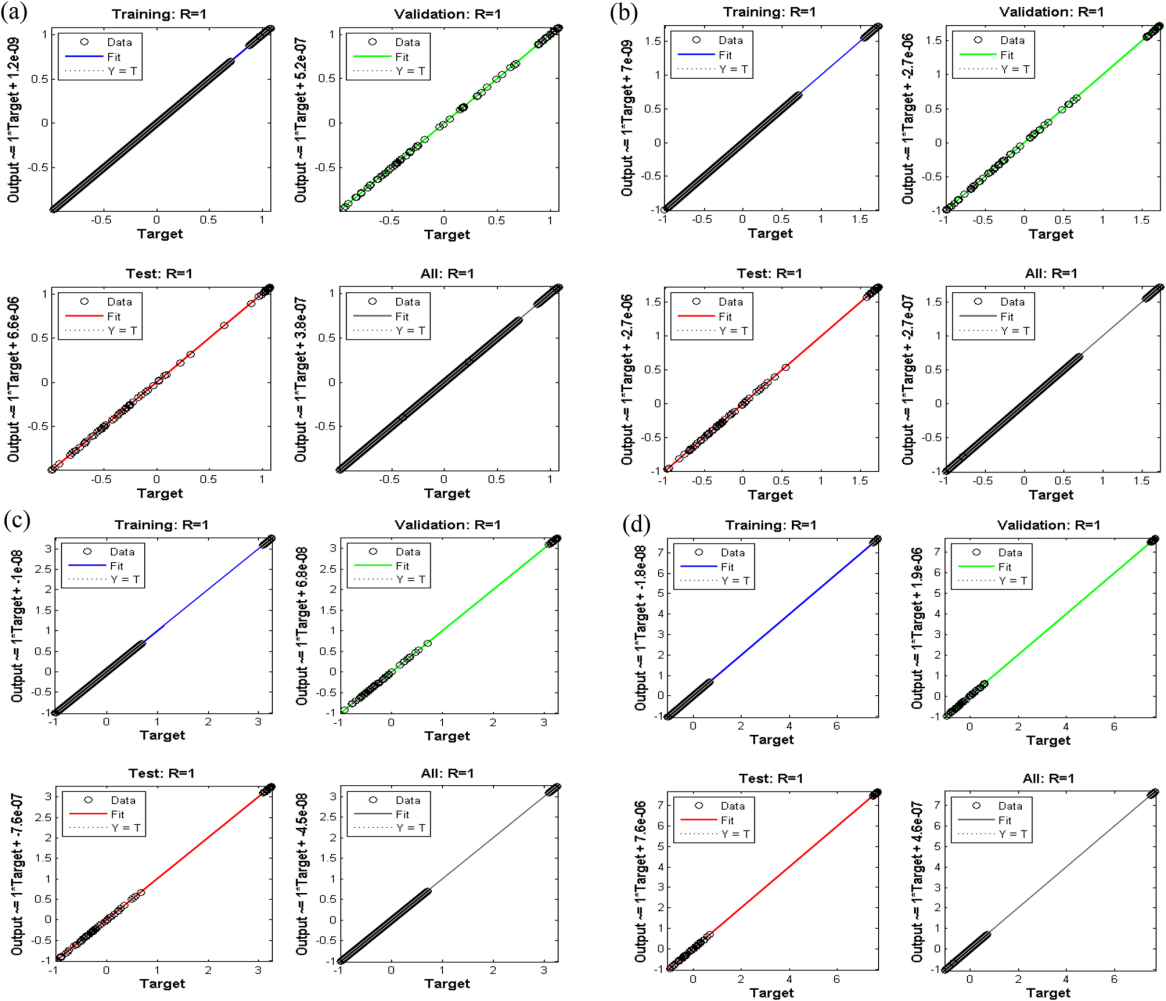
Regression plots of case study 1: scenario 1 (**a**) case 1, (**b**) case 2, (**c**) case 3 and (**d**) case 4.

**Figure 13:**
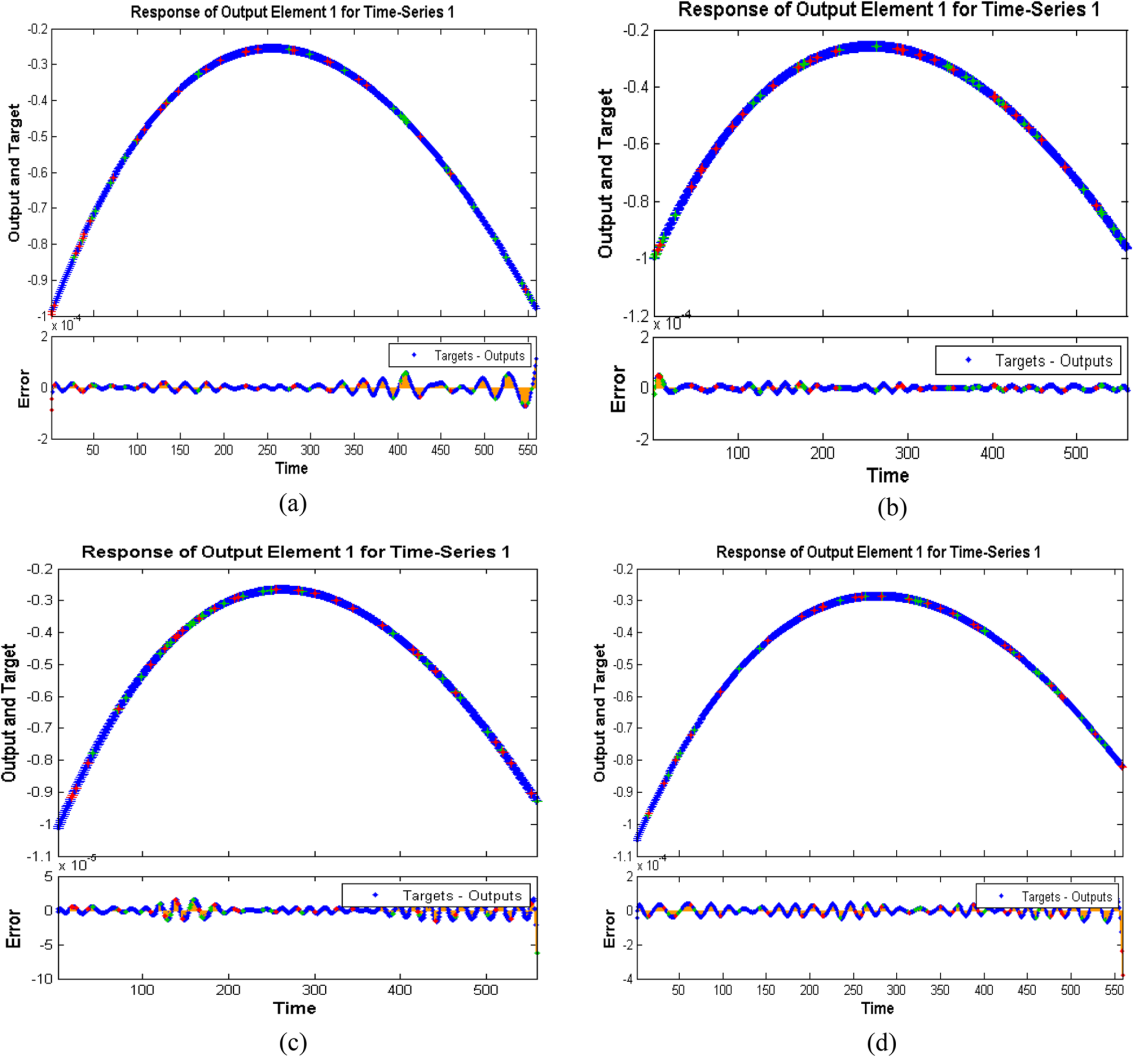
Time series response of case study 1: scenario 1 (**a**) case 1, (**b**) case 2, (**c**) case 3 and (**d**) case 4.

**Figure 14:**
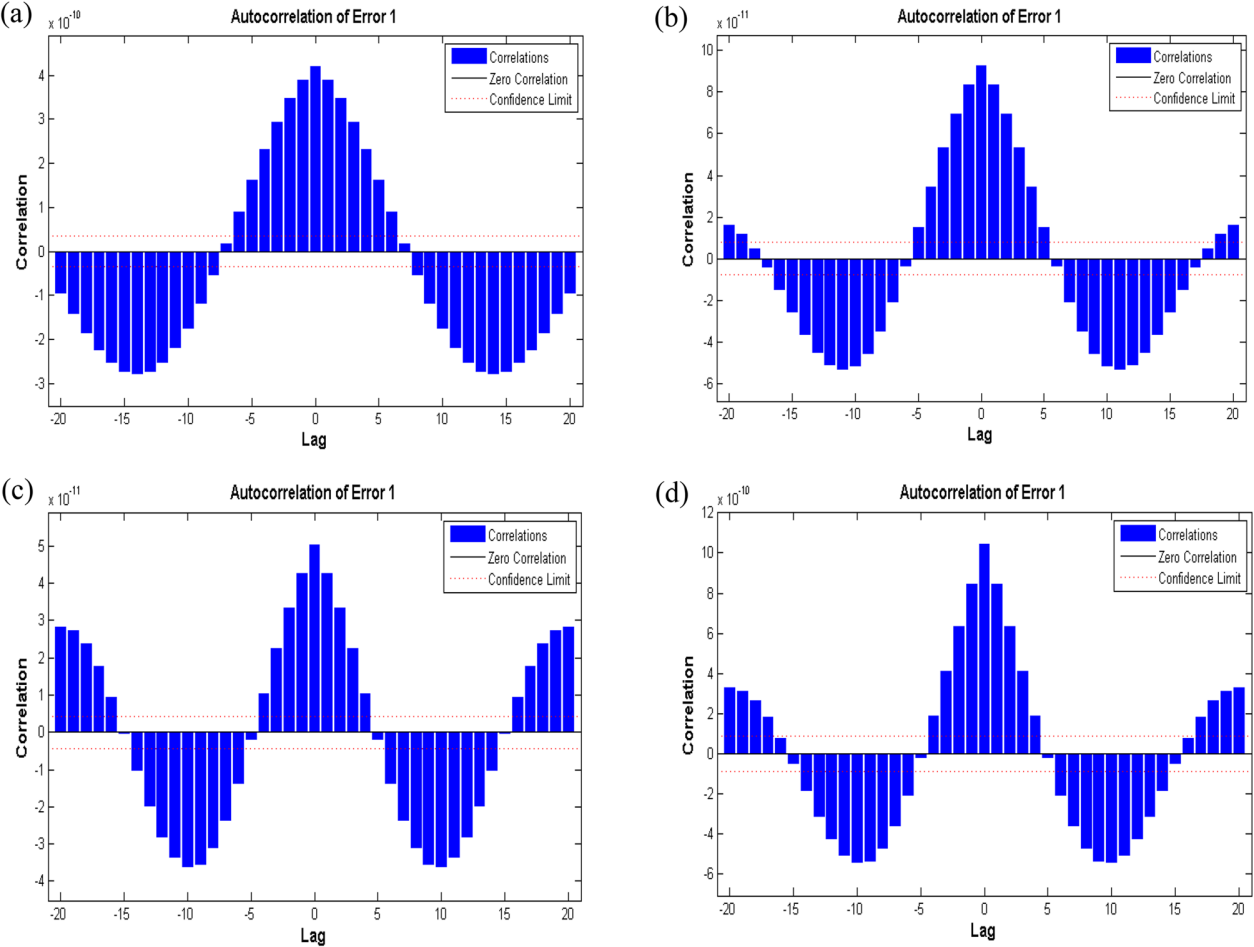
Auto-correlation plots of case study 1: scenario 1 (**a**) case 1, (**b**) case 2, (**c**) case 3 and (**d**) case 4.

Performance analysis for 4 cases of the all 3 scenarios namely *ϕ*_2,_ *β*_1_ and *β*_2_, for a reliable prediction of velocity profile is displayed in Figs. 9, 10, 11, 12, 13 and 14.

Figure 9a–d displays the network performance in terms of mean square error against epochs curve. It is depicted that the trained data is very accurately validated for all cases of scenario 1 with the best performance at epochs 190, 257, 343 and 211 corresponding to least mean square error of order e^−10^, respectively. Figure 10a–d illustrate gradient value, mu parameter along with validation checks instance. It is witnessed that gradient and mu are locally decreasing functions against iterations i.e., error is reducing for increasing epochs, whereas validation check is constant. Error histograms in Fig. 11a–d for different cases of scenario 1 represent that the error between the target and output of network is very close to zero. Moreover, the positive difference is comparably lesser than negative difference for case 1 and case 4, while contrary trend is shown for cases 2 and 3.

Figure 12a–d represents regression plots for scenario 1. Since, R be the correlation for the outputs with targets and should be closed to R = 1 and avoid random scenario of R = 0; regression plots of all four cases show that data is highly correlated and concentrated i.e., R = 1. It is also noteworthy that regression plot for case 1 of scenario 1 is highly efficient due to small empty space. The empty space is because of missing values in data.

Numerical simulations are further summarized by plotting time series response for target and output data in Fig. 13a–d. In present study, ‘ton data’ time series format is used to arrange the data according to standard network cell array form. There is a specific interval for each case of scenario 1 which contains both output and target and the error for each time step of data is presented up to total time steps i.e., 561. This means that the analysis predicts the proposed results for training and testing data with certain accuracy, i.e., reasonable precision, but error performance is relatively poor for testing from training. Further, the results are validated with auto-correlation plots in Fig. 14a–d. It is depicted that error autocorrelation is smaller for case 3 of scenario 1. The similar trends for neural network results for different cases of scenario 2 and scenario 3 are observed.

#### Case study 2

**Figure 15:**
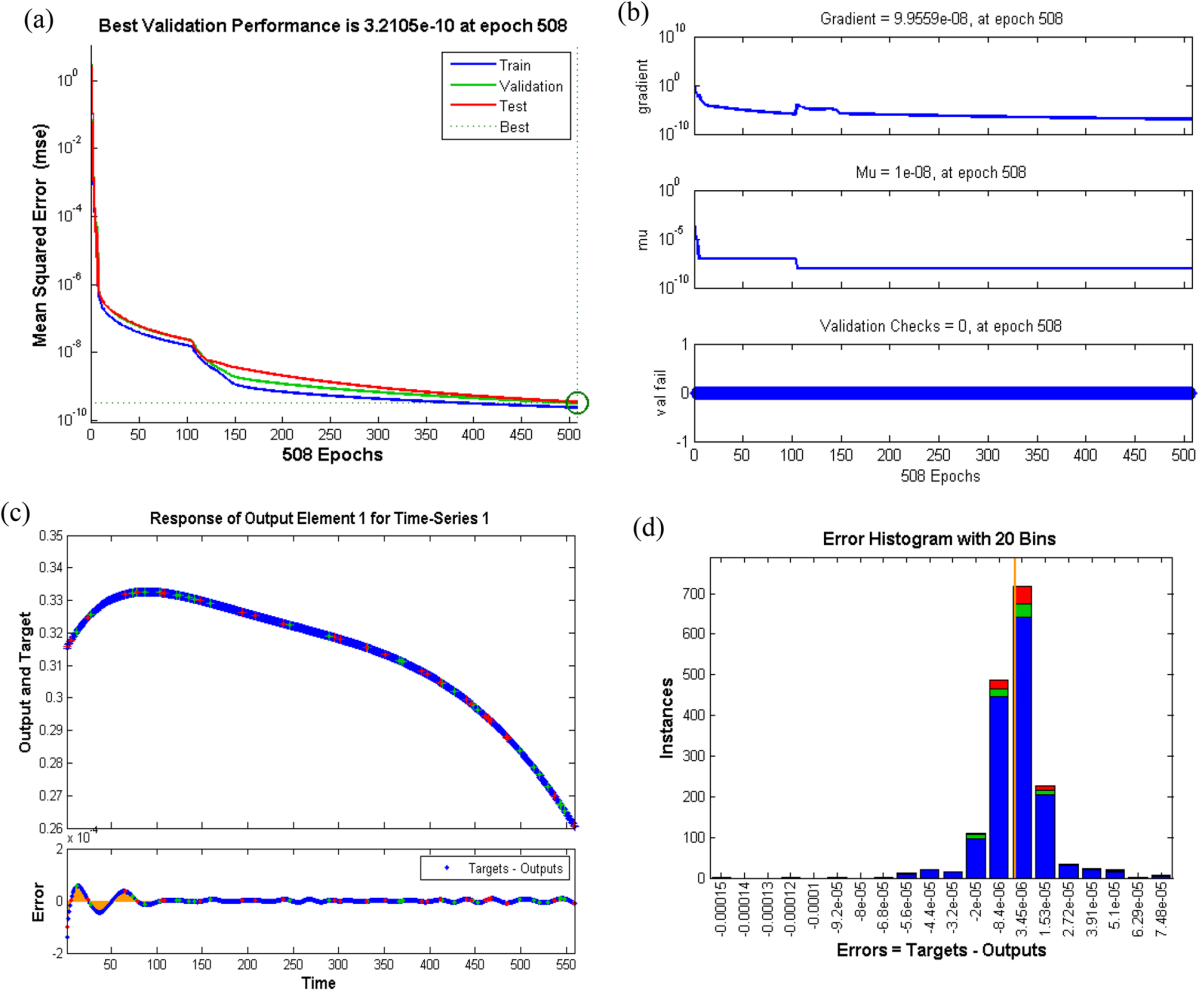
Performance curve of case study 2: scenario1 (**a**) learning curves of case 1, (**b**) training state of case 1, scenario 2, (**c**) time series response of case 1 and (**d**) error histogram for case 3.

The analysis for neural network to analyze the impact of temperature on hybrid nanofluid for the two scenarios with several cases is provided in Table 1. Performance of the network is plotted in Fig. 15. In case of MSE versus epochs and it is shown that best validation performance is achieved for increasing number of iterations for both scenarios. Moreover, validated results for case 2 of scenario 1 are more efficient than case 1 with performance at 2.7656e^−10^ at epochs 324, while case 3 of scenario 2 is more accurate than case 1 with validation performance 4.3012e^−11^ at epochs 284. In case of training state plots, it can be inferred that gradient parameter and mu factor are reducing with increase in number of iterations. These states are further befitted by accurate displaying of error histogram. Additionally, Most of the errors against components of neural networks i.e. training, validation and testing phases approach zero. Eventually, for given time steps of dataset, results are validated by plotting time series response. It is evident that error for each case of the two scenarios is very close to zero error in given range for corresponding epochs which shows an accurate prediction.

Experimental values of thermophysical features of base fluid and hybrid nanoparticles are expressed in Table 2. Mathematical relations for thermophysical properties of hybrid nanomaterials are also represented in Table 3.

**Table 2:** Experimental values of thermal features of nanomaterials and base fluid at 25 °C.

Properties\constituents	H_2_O	MWCNTs	Au
Density, ρ (kg/m^3^)	997.1	1600	19,300
Specific heat, C_p_ (J/kg K)	4179	796	129
Thermal conductivity, κ (W/m K)	0.613	3000	318

**Table 3:** Expressions for thermophysical characteristics of hybrid nanofluid.

Properties	Hybrid nanofluid
Density	$\rho_{{{\text{hnf}}}} = \rho_{{\text{f}}} \left( {1 - \varphi_{2} } \right)\left[ {\left( {1 - \varphi_{1} } \right) + \varphi_{1} \left( {\frac{{\rho_{{{\text{s}}_{1} }} }}{{\rho_{{\text{f}}} }}} \right)} \right] + \varphi_{2} \rho_{{{\text{s}}_{2} }}$
Heat capacity	$\left( {\rho {\text{c}}_{{\text{p}}} } \right)_{{{\text{hnf}}}} = \left( {\rho {\text{c}}_{{\text{p}}} } \right)_{{\text{f}}} \left( {1 - \varphi_{2} } \right)\left[ {\left( {1 - \varphi_{1} } \right) + \varphi_{1} \left( {\frac{{\left( {\rho {\text{c}}_{{\text{p}}} } \right)_{{{\text{s}}_{1} }} }}{{\left( {\rho {\text{c}}_{{\text{p}}} } \right)_{{\text{f}}} }}} \right)} \right] + \varphi_{2} \left( {\rho {\text{c}}_{{\text{p}}} } \right)_{{{\text{s}}_{2} }}$
Viscosity	$\mu_{{{\text{hnf}}}} = \frac{{\mu_{{\text{f}}} }}{{\left( {1 - \varphi_{1} } \right)^{2.5} \left( {1 - \varphi_{2} } \right)^{2.5} }}$
Thermal conductivity	$$\begin{aligned} & \frac{{\kappa_{{{\text{hnf}}}} }}{{\kappa_{{{\text{bf}}}} }} = \frac{{\kappa_{{{\text{s}}_{2} }} + \left( {{\text{s}} - 1} \right)\kappa_{{{\text{bf}}}} - \left( {{\text{s}} - 1} \right)\varphi_{2} \left( {\kappa_{{{\text{bf}}}} - \kappa_{{{\text{s}}_{2} }} } \right)}}{{\kappa_{{{\text{s}}_{2} }} + \left( {{\text{s}} - 1} \right)\kappa_{{{\text{bf}}}} + \varphi_{2} \left( {\kappa_{{{\text{bf}}}} - \kappa_{{{\text{s}}_{2} }} } \right)}}, \\ & {\text{where}}\,\,\frac{{\kappa_{{{\text{bf}}}} }}{{\kappa_{{\text{f}}} }} = \frac{{\kappa_{{{\text{s}}_{1} }} + \left( {{\text{s}} - 1} \right)\kappa_{{\text{f}}} - \left( {{\text{s}} - 1} \right)\varphi_{1} \left( {\kappa_{{\text{f}}} - \kappa_{{{\text{s}}_{1} }} } \right)}}{{\kappa_{{{\text{s}}_{1} }} + \left( {{\text{s}} - 1} \right)\kappa_{{\text{f}}} + \varphi_{1} \left( {\kappa_{{\text{f}}} - \kappa_{{{\text{s}}_{1} }} } \right)}} \\ \end{aligned}$$
Thermal expansion coefficient	$(\rho \beta )_{{{\text{hnf}}}} = (\rho \beta )_{{\text{f}}} \left[ {(1 - \phi_{1} - \phi_{2} ) + \phi_{1} \left( {\frac{{(\rho \beta )_{{{\text{s}}_{1} }} }}{{(\rho \beta )_{{\text{f}}} }}} \right)} \right] + \phi_{2} (\rho \beta )_{{{\text{s}}_{2} }}$

Moreover, the details values of the parameters while execution on neural networks in terms of selected number of neurons in the architecture, observed epochs, values of MSE, R value, errors for gradient index and mu parameter for respective cases of each scenario of the two case studies are tabulated in Tables 4 and 5, respectively. This tabular representation is useful in verification and validation of results of the proposed neural network based stochastic solver. It is noted that *R* value in each case is 1 which is an indicative of good fit. Except this, numerical results found by Adams Bashforth technique for stream function, velocity and temperature for corresponding interval [− 1.4, 1.4] with step size 0.1 are represented in Table 6 for fixed values of *ϕ*_2_ = 0.02, *β*_1_ = *β*_2_ = 0.01, *k* = 5.0, F = − 1.4 and Bi = 0.5, which satisfy the results.

**Table 4:** Comparative analysis for all four cases of three scenarios of case study 1.

Scenario number	Case index	Neurons settings	Fitness on MSE			Value of gradient	Value of performance	R	Epochs index	mu parameter
			Training	Validation	Testing					
1	1	50	2.3961e−10	4.1839e−10	6.5676e−10	9.9826e−08	4.1839e−10	1	190	1.00e−09
	2	50	7.8543e−11	1.3970e−10	1.3721e−10	9.9681e−08	1.3970e−10	1	257	1.00e−09
	3	50	6.0199e−11	1.5856e−10	6.1410e−11	9.8812e−08	1.5856e−10	1	343	1.00e−09
	4	80	4.9520e−10	6.2145e−10	4.3162e−09	9.9899e−08	6.2145e−10	1	211	1.00e−08
2	1	80	4.3322e−11	1.6575e−10	5.0701e−11	9.9724e−08	1.6575e−10	1	214	1.00e−09
	2	70	2.1824e−10	7.0135e−10	1.9393e−10	9.9452e−08	4.7605e−11	1	310	1.00e−09
	3	70	1.4602e−11	1.8643e−11	5.5134e−11	9.9963e−08	1.8643e−11	1	563	1.00e−09
	4	80	4.0620e−11	4.7605e−11	1.7691e−10	9.9945e−08	2.5030e−10	1	502	1.00e−09
3	1	50	4.5758e−11	1.8526e−10	6.4113e−11	9.9388e−08	1.2851e−10	1	294	1.00e−08
	2	50	2.1670e−10	2.8708e−10	3.2116e−10	9.9220e−08	2.8708e−10	1	357	1.00e−08
	3	80	3.4791e−11	5.1742e−11	1.333e−10	9.9182e−08	5.1742e−11	1	315	1.00e−09
	4	50	2.6416e−11	2.0837e−11	6.7688e−11	9.9917e−08	2.0837e−11	1	497	1.00e−09

**Table 5:** Comparative Analysis for all four cases of two scenarios of case study 2.

Scenario number	Case index	Neurons settings	Fitness on MSE			Value of gradient	Value of performance	R	Epochs index	mu parameter
			Training	Validation	Testing					
1	1	50	8.8625e−11	4.3316e−10	8.9608e−11	9.9836e−08	4.3316e−10	1	322	1.00e−09
	2	65	1.9807e−10	1.9603e−10	2.8823e−10	9.9874e−08	1.9603e−10	1	365	1.00e−08
	3	50	4.0828e−11	4.3012e−11	6.5085e−11	9.9194e−08	4.3012e−11	1	284	1.00e−09
	4	50	7.6003e−11	3.6519e−10	7.5976e−11	9.8325e−08	3.6519e−10	1	216	1.00e−09
2	1	60	2.3969e−10	3.2105e−10	3.4920e−10	9.9559e−08	3.2105e−10	1	508	1.00e−08
	2	70	2.5292e−10	2.7656e−10	2.3016e−10	9.9740e−08	2.7656e−10	1	324	1.00e−08
	3	60	1.9040e−10	1.9361e−10	2.7741e−10	9.9700e−08	1.9361e−10	1	374	1.00e−08
	4	50	9.8250e−11	4.6765e−10	1.0185e−10	9.8898e−08	4.6765e−10	1	327	1.00e−09

**Table 6:** Variation in magnitude of flow variables for different values of *R.*

r	$\psi$	$u$	$\theta$
− 1.4	− 0.7	− 1.0086666978	− 1.0737437912
− 1.3	− 0.6054760383	− 0.8841129360	1.0953057138
− 1.2	− 0.522117340	− 0.7729501422	1.1081789287
− 1.1	0.4504653796	− 0.6744085801	1.1148446570
− 1	− 0.3874521123	− 0.5877888553	1.1171845075
− 0.9	− 0.3325315182	− 0.5124536895	1.116095763
− 0.8	− 0.2846046841	− 0.4478208613	1.1141616614
− 0.7	− 0.2426284281	− 0.3933570210	1.1105928826
− 0.6	− 0.2056106828	− 0.3485723973	1.1064284445
− 0.5	− 0.1726063737	− 0.3130161856	1.1020162554
− 0.4	− 0.142712954	− 0.286275194	1.0975662454
− 0.3	− 0.1150709705	− 0.2679568449	1.0931814743
− 0.2	− 0.0888533271	− 0.2577130328	1.0888829859
− 0.1	− 0.0632703498	− 0.2552104115	1.0846293649
0.0	− 0.0375634784	− 0.2601414902	1.0803324058
0.1	− 0.0110038326	− 0.2722197821	1.0758694915
0.2	0.0171097868	− 0.2911779084	1.0710934221
0.3	0.0474527271	− 0.3167659078	1.0658401839
0.4	0.0806761461	− 0.3487479267	1.0599350762
0.5	0.1174085351	− 0.3869098699	1.0531975192
0.6	0.1582571124	− 0.4310401871	1.0454448049
0.7	0.2038091028	− 0.4809467885	1.0364949996
0.8	0.2546329141	− 0.5364470606	1.0261691717
0.9	0.3112792193	− 0.5973687852	1.0142930625
1.0	0.3742819556	− 0.6635493391	1.0006983327
1.1	0.4441592473	− 0.7348349704	0.9852234530
1.2	0.5214142593	− 0.8110801396	0.9677143119
1.3	0.6065359879	− 0.8921469243	0.9480264723
1.4	0.6999999957	− 0.9779044746	0.9260161893

Figures 1 and 2 are created using Microsoft 365 Apps for enterprise, Version 2105 (Build 14026,20246) , https://pcp.pern.edu.pk while the simulation studies are performed using Matlab 2021a, License ID 40727596, https://www.mathworks.com/products/matlab.html. and Mathematica 12, License ID L4469-7058, Activation Key: 4469-7058-66XHRH. https://www.wolfram.com/mathematica.

## Conclusions

A novel application of intelligent computing framework mainly based on neural networks is portrayed for solving a model arises in nanotechnology field with bio-medical and geometrical features. Accuracy is validated through achievement of MSE of the order of 10^−10^ or 10^−11^. An effective correlation observed for target with outputs having value close to *R* = 1, a decreasing trend in gradient index with error near zero. In addition, time series response is shows consistent overlapping of results with reference solutions, and the error remained close to 0 error line for each scenario of the system model. Experimental observations show the accuracy, robustness and stability of the proposed computing frameworks, i.e., the neural network paradigm trained with Levenberg–Marquart algorithm. It is also proved that neural network is a powerful tool for analysis and network outputs show a reasonable accuracy for flow variables against different cases of each scenario of the system model.

Moreover, the problem is physically imperative concerning its geometrical features as several physiological vessels are curved in nature. Present attempt is appropriate in medical field to estimate and get the best possible outcomes for the effectiveness of nanomaterials, and generally to identify patterns in different scientific divisions by employing neural network algorithm. This is evidently a developing field and future attempts with fractional evolutionary/swarming based optimization algorithms^65–70^ look promising for better outcomes and to enhance the neural network performance considerably.
